# Evaluation and optimization of PCR primers for selective and quantitative detection of marine ANME subclusters involved in sulfate-dependent anaerobic methane oxidation

**DOI:** 10.1007/s00253-017-8338-x

**Published:** 2017-06-15

**Authors:** Peer H. A. Timmers, H. C. Aura Widjaja-Greefkes, Caroline M. Plugge, Alfons J. M. Stams

**Affiliations:** 1 Laboratory of Microbiology, Wageningen University, Stippeneng 4, 6708 WE Wageningen, the Netherlands; 2 Centre of Excellence for Sustainable Water Technology, Wetsus, Oostergoweg 9, 8911 MA Leeuwarden, the Netherlands; 3 Centre of Biological Engineering, University of Minho, Campus de Gualtar, 4710-057 Braga, Portugal

**Keywords:** Anaerobic oxidation of methane, AOM, Methanotrophs, ANME, qPCR, Primers

## Abstract

Since the discovery that anaerobic methanotrophic archaea (ANME) are involved in the anaerobic oxidation of methane coupled to sulfate reduction in marine sediments, different primers and probes specifically targeting the 16S rRNA gene of these archaea have been developed. Microbial investigation of the different ANME subtypes (ANME-1; ANME-2a, b, and c; and ANME-3) was mainly done in sediments where specific subtypes of ANME were highly enriched and methanogenic cell numbers were low. In different sediments with higher archaeal diversity and abundance, it is important that primers and probes targeting different ANME subtypes are very specific and do not detect other ANME subtypes or methanogens that are also present. In this study, primers and probes that were regularly used in AOM studies were tested in silico on coverage and specificity. Most of the previously developed primers and probes were not specific for the ANME subtypes, thereby not reflecting the actual ANME population in complex samples. Selected primers that showed good coverage and high specificity for the subclades ANME-1, ANME-2a/b, and ANME-2c were thoroughly validated using quantitative polymerase chain reaction (qPCR). From these qPCR tests, only certain combinations seemed suitable for selective amplification. After optimization of these primer sets, we obtained valid primer combinations for the selective detection and quantification of ANME-1, ANME-2a/b, and ANME-2c in samples where different ANME subtypes and possibly methanogens could be present. As a result of this work, we propose a standard workflow to facilitate selection of suitable primers for qPCR experiments on novel environmental samples.

**Electronic supplementary material:**

The online version of this article (doi:10.1007/s00253-017-8338-x) contains supplementary material, which is available to authorized users.

## Introduction

**Fig. 1:**
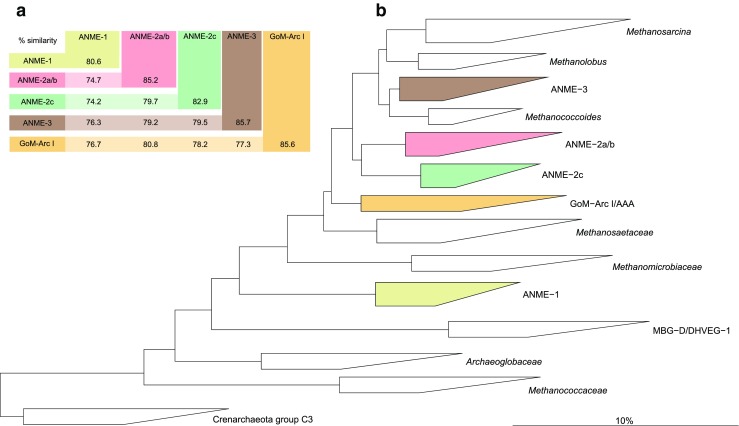
**a** The lowest percentage of 16S rRNA gene sequence similarity between and within ANME clades and the GoM-Arc I clade (that contained ANME-2d). Similarities were calculated using all sequences of the specific clades from the SILVA 16S rRNA database version SSU r122 Ref NR (Quast et al. 2013) with the distance matrix method of the ARB software package with similarity correction (Ludwig et al. 2004). **b** Phylogenetic tree of full length 16S rRNA gene sequences of archaeal clades that harbor AOM performing archaea (*colored*) and other non-AOM performing clades (*white*). Using 1291 sequences from the SILVA SSUref NR 99 database (release 119.1) (Quast et al. 2013), the tree was constructed with the ARB software package (version arb-6.0.1.rev12565) (Ludwig et al. 2004). Trees (bootstrapping value of 1000 trees) were calculated with the ARB neighbor-joining method with terminal filtering and the Jukes-Cantor correction. Crenarchaeota group C3 was used as outgroup. The *scale bar* represents the percentage of changes per nucleotide position

Atmospheric methane (CH_4_) is the second most important greenhouse gas on earth and accounts for 20% of all the infrared radiation captured in the atmosphere (Dale et al. 2006). Marine sediments produce significant amounts of methane, and most methane derives from organic matter degradation and to a lesser extent from thermogenic and geochemical processes (Reeburgh 2007; Thauer and Shima 2008). The produced methane only partly reaches the water column through seeps, vents, and mud volcanoes or via diffusion from anoxic sediments and dissolution of methane clathrate hydrates. More than 90% of the annually produced methane is oxidized coupled to sulfate reduction (SR) in anoxic marine sediments before it reaches the hydrosphere (reviewed in Hinrichs and Boetius 2002; Knittel and Boetius 2009; Reeburgh 2007). Anaerobic oxidation of methane (AOM) coupled to SR was first discovered in marine sediments at the zone where gradients of methane and sulfate overlap, the sulfate-methane transition zone (SMTZ) (Martens and Berner 1974; Reeburgh 1976). Molecular studies showed that most archaeal 16S rRNA gene sequences that were retrieved from marine methane-oxidizing environments belonged to specific clades in the *Euryarchaeota* that were named anaerobic methanotrophic archaea (ANME) (Hinrichs et al. 1999; Boetius et al. 2000; Orphan et al. 2001). In marine environments, three clades of ANME were identified and these were named ANME-1 (consisting of subclusters a and b), ANME-2 (consisting of subclusters a, b, and c), and ANME-3. The ANME-1 cluster is related to *Methanomicrobiales* and *Methanosarcinales* but forms a separate cluster (Hinrichs et al. 1999), ANME-2 are related to cultivated members of the *Methanosarcinales* (Hinrichs and Boetius 2002), and ANME-3 are most related to *Methanococcoides* spp. (Knittel et al. 2005). The subclusters ANME-2a and ANME-2b were subdivided, but they form a coherent clade that is clearly separated from ANME-2c, and they are therefore often clustered as ANME-2a/b (Timmers et al. 2017) (Fig. 1). The sequences derived from clone libraries of the first studies were used to develop probes for fluorescence in situ hybridization (FISH) and primers for quantitative PCR (qPCR)-based analysis. These probes and primers were mainly used to study seep systems and microbial mats where the in situ archaeal community was investigated using 16S rRNA gene analysis. The majority of these previously developed widely used probes and primers such as EelMS932 (Boetius et al. 2000), ANME-1-350 (Boetius et al. 2000), ANME2a-647, ANME-2c-622, and ANME-2c-760 (Knittel et al. 2005) were indeed suitable to study archaea involved in AOM in these environments. However, it is not known if these probes and primers capture the full diversity within ANME clades and if they are specific for certain ANME clades that occur in other environments. The ANME-3 subtype has so far only been reported to occur in some mud volcanoes (Lösekann et al. 2007; Niemann et al. 2006; Omoregie et al. 2008) whereas in most marine sediments, the ANME subtypes ANME-1, ANME-2a, ANME-2b, and ANME-2c and different methanogens are present and show overlapping regions of occurrence (Nunoura et al. 2006; Orcutt et al. 2005; Orphan et al. 2004; Pachiadaki et al. 2011; Roalkvam et al. 2011, 2012; Yanagawa et al. 2011). Therefore, in different marine sediments that harbor a high diversity of ANME and methanogens, it is important that primers and probes targeting ANME are very specific and do not detect other ANME subtypes or methanogens that are present. It indeed appeared that published primer pairs and probes were less suited for other environments, especially in quantitative PCR (qPCR) experiments. Thus, new specific primers emerged, but the design, validation and optimization of primers for the different ANME subclades is difficult. This is mainly because the phylogenetic distances are large between ANME subclades as well as within ANME subclades (Knittel and Boetius 2009). With more 16S rRNA gene sequences emerging in the database, primers and probes are continuously developed to detect novel ANME sequences, or when published ones were deemed not specific.

**Fig. 2:**
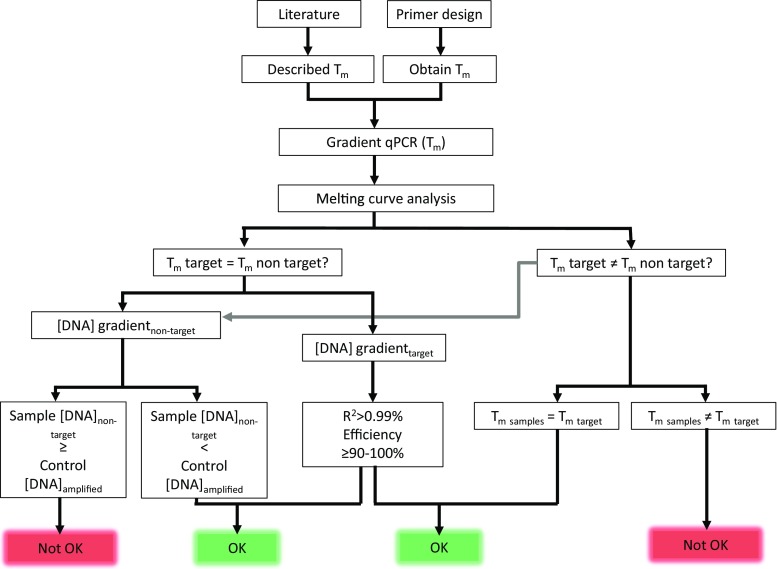
Flowchart of qPCR approach when existing or newly designed primers are used with a complex AOM sample

In this study, we performed in silico validation of the so far published primers and probes that were used to study ANME that performed sulfate-dependent AOM in marine sediments. We therefore focussed on oligonucleotides that target the clusters ANME-1, ANME-2a/b (previous primer sets covering only ANME-2a or only ANME-2b were not tested in this work), and ANME-2c. For each probe or primer pair, we studied the coverage of the target ANME groups, as well as the coverage of non-target groups. When oligonucleotides seemed suitable, in vitro validation and optimization was done for specific amplification of ANME-1, ANME-2a/b, and ANME-2c, using quantitative PCR. Validation of primers was done using cloned full-length 16S rRNA gene sequence inserts of ANME-1, ANME-2a/b, and ANME-2c archaea, as well as 16S rRNA gene sequence inserts of *Methanococcoides* sp. and genomic DNA from *Methanosarcina mazei* strain MC3 and *Desulfovibrio* G11. We also included environmental samples from Eckernförde Bay (Baltic Sea, Denmark) which is a gassy diffusive sediment different from seeps and hydrothermal vents, since methane is produced from in situ organic matter degradation (Treude et al. 2005b). This sediment contained ANME-1, ANME-2a/b, and ANME-2c and methanogens (Timmers et al. 2015a, b) and is therefore highly suitable to validate primers and probes on specificity for the different ANME subtypes. High specificity will enable studies on abundance and occurrence of different ANME clades which is important for understanding global methane emissions from marine sediments and other methane-cycling environments. The workflow applied for evaluation and optimization of qPCR primer sets is shown in Fig. 2.

## Materials and methods

### In silico testing of probes and primers

Reported probes and primers used in marine AOM studies were tested for coverage and specificity, using the SILVA Probe Match and Evaluation Tool - TestProbe 3.0 and Testprime 1.0 services (Klindworth et al. 2013) with the SILVA 16S rRNA database version SSU r128 Ref NR (Quast et al. 2013). Only results with 100% specificity (0 mismatches) were used for both probes and primers. Primer pairs that were a mixture of multiple forward or reverse primers were submitted with a degenerate base to Testprime 1.0. For instance, primer ANME1-395F consists of a mixture of three different primers and ANME1-1417R consisted of a mixture of two different primers for increasing coverage of the target ANME-1 group (Miyashita et al. 2009). Therefore, we combined a maximum of two primers in each Testprime submission by replacing one base with the degenerate base that covers both primers; in this case we submitted ANME1-395F (1 + 2)/ANME1-1417R (1 + 2) and ANME1-395F (3)/ANME1-1417R (1 + 2) to Testprime (see sequence information of the primers in Table 1). This results in a different coverage than when all three primers in this in vitro PCR were combined. Primer and probe coverage of target and non-target groups is given in Tables 1 and 2, respectively.

**Table 1:** Primers described in the literature that were used for ANME detection

Primer combination	Sequence	Target	Coverage (%)	Non-target	Coverage (%)	Product size (bp)	Reference
^**a**^ **ANME1-395F (1+2) /** **ANME1-1417R (1+2)**	AAC TCT GAG TGC CTC CWA / CCT CAC CTA AAY CCC ACT	ANME-1	62.2	*Methanomicrobia* (other) *Euryarchaeota* (other)	3.4 1.0	1039	(Miyashita et al. 2009)
^**a**^ **ANME1-395F (3) /** **ANME1-1417R (1+2)**	AAC TCT GAG TGC CCC CTA / CCT CAC CTA AAY CCC ACT	ANME-1	2.7	*Methanomicrobia* (other)	0.1	1039	(Miyashita et al. 2009)
**ANME-1-337F /** **ANME-1-724R**	AGG TCC TAC GGG ACG CAT / GGT CAG ACG CCT TCG CT	ANME-1	68.6	*Methanomicrobia* (other) *Euryarchaeota* (other)	3.3 0.9	358	(Girguis et al. 2005)
ANME1-628f / ANME1-830r	GCT TTC AGG GAA TAC TGC / TCG CAG TAA TGC CAA CAC	ANME-1	40	*Methanomicrobia* (other) *Euryarchaeota* (other)	1.9 0.5	219	(Lloyd et al. 2011) (Boetius et al. 2000)
^**a**^ **ANME2a -426F (1+2) /** **ANME2a-1242R**	TGT TGG CTG TCC RGA TGA / AGG TGC CCA TTG TCC CAA	ANME-2a/b	38.9	*Methanosarcinales* (other)	3.3	833	(Miyashita et al. 2009)
				*Methanomicrobia* (other) *Euryarchaeota* (other)	1.9 0.4		
^**a**^ **ANME-2a-426F (3) /** **ANME2a-1242R**	TGT TGG CTG TCC AGA TGG / AGG TGC CCA TTG TCC CAA	ANME-2a/b	9.5	*Methanosarcinales* (other)	0.8	833	(Miyashita et al. 2009)
				*Methanomicrobia* (other) *Euryarchaeota* (other)	0.5 0.1		
ANME-2aF / ANME-2aR	ACG GAT ACG GGT TGT GAG AG / CTT GTC TCA GTC CCC GTC TC	-	0	-	0	-	(Vigneron et al. 2013)
ANME2b-402F / ANME2b-1251R	AGT GCC AGT ACT AAG TGC / TTT CGA GGT AGG TAC CCA	ANME-2b	42.9	*Methanosarcinales* (other)	0.3	866	(Miyashita et al. 2009)
		ANME-2a/b	3.2	*Methanomicrobia* (other)	0.2		
^**a**^ **ANME2c-AR468F (1+2) / ANME2c-AR-1411R**	CGC RCA AGA TAG CAA GGG / CCA AAC CTC ACT CAG ATG	ANME-2c	48.3	*Methanosarcinales* (other)	2.6	960	(Miyashita et al. 2009)
				*Methanomicrobia* (other) *Euryarchaeota* (other)	1.5 0.4		
^a^ **ANME2c-AR468F (3) /** **ANME2c-AR-1411R**	AGC ACA AGA TAG CAA GGG / CCA AAC CTC ACT CAG ATG	ANME-2c	18.6	*Methanosarcinales* (other)	1.0	960	(Miyashita et al. 2009)
				*Methanomicrobia* (other) *Euryarchaeota* (other)	0.6 0.2		
**ANME2c-F /** **ANME-2c-R**	TCG TTT ACG GCT GGG ACT AC / TCC TCT GGG AAA TCT GGT TG	ANME-2c	65.6	*Methanosarcinales* (other)	3.2	221	(Vigneron et al. 2013)
				*Methanomicrobia* (other)	1.8		
	(F and R are switched)			ANME-2a/b *Euryarchaeota* (other)	1.6 0.5		
**Ar-468F /** **AR736R**	CGC ACA AGA TAG CAA GGG / CGT CAG ACC CGT TCT GGT A	ANME-2c	60.2	*Methanosarcinales* (other)	2.8	268	(Girguis et al. 2003)
				*Methanomicrobia* (other) *Euryarchaeota* (other)	1.6 0.4		
ANMEF / 907R	GGCUCAGUAACACGUGGA / CCGTCAATTCCTTTRAGTTT	ANME-3	1.6	pMC2A209	25	816	(Thomsen et al. 2001)
				*Methanosarcinales* (other)	0.2		
				*Desulfuromonadales*	0.1-0.3		
				*Lokiarchaeota*	0.1		
				MBG-D	0.1		

All primer combinations were tested using the online Testprime database of SILVA, using 100% specificity (0 mismatches). Primers tested in this study are displayed in bold.

^a^These primers are a mixture of separately designed primers (indicated by number in brackets) as described by Miyashita et al. (2009), see “Materials and methods” for explanation

**Table 2:** Probes described in the literature that were used for ANME detection

Probe name	Sequence (5’-3’)	Target site	Target	Coverage	Non-target	Coverage	Reference
ANME-1-305	AGC CCG GAG ATG GGT TCT	305-322	ANME-1	67.9	*Methanocellales*	2-2.8	(Boetius et al. 2000)
					*Thermoplasmatales*	1.2-100	
					*Thaumarchaeota*	0.1	
					*Methanosarcinales* (other)	0.2-0.3	
					*Aenigmarchaeota*	0.4-0.7	
					Candidate division YNPFFA	23.9	
					GOM Arc I	0.9	
ANME-1-350	AGT TTT CGC GCC TGA TGC	350-367	ANME-1	91.6	*Thaumarchaeota*	0.8-26.9	(Boetius et al. 2000)
					*Crenarchaeota*	0.2-4.3	
					*Methanomicrobiaceae*	0.2-0.4	
					*Methanosarcinales* (other)	0.1	
					*Thermoplasmata*	0.1-0.2	
					*Hadesarchaea*	0.6	
					Class WSA2	0.5-1.5	
ANME1-632	TCA GGG AAT ACT GCT TGG	632-649	ANME-1	50.3	*Methanomicrobia* (other)	2.4	(Boetius et al. 2000)
ANME-1-862	GGC GGG CTT AAC GGG CTT C	86-880	-		*Sulfolobales*	0.5-5	(Orphan et al. 2002)
					*Crenarchaeota*	0.2	
ANME1-830	TCG CAG TAA TGC CAA CAC	830-847	ANME-1	75.9	*Methanomicrobia* (other)	3.6	(Boetius et al. 2000)
ANME-2-538	GGC TAC CAC TCG GGC CGC	538–555	ANME-2c	78.3	GOM Arc I	55.7	(Treude et al., 2005a)
					*Ca.* Methanoperedens	68.7	
			ANME-2a/b	63.1	*Methanosarcinales* (other)	0.1-31.6	
					*Methanococci*	2.5-8.3	
			ANME-2b	12.5	MSBL-1	9.1	
					*Methanomicrobia* (other)	7.6	
					*Euryarchaeota* (other)	2.0	
ANME-2-712	TTC GCC ACA GAT GGT CCC	712-729	ANME-2a/b	89.3	*Methanosarcinales* (other)	0.4-15.8	(Knittel and Boetius 2009)
			ANME-2c	79.8	*Methanomicrobia* (other)	5.8	
			ANME-2b	87.5	*Aenigmarchaeota*	1-1.2	
					WSA2	0.9-2.5	
ANME2a-647	TCT TCC GGT CCC AAG CCT	647-664	ANME-2a/b	62.3	*Methanosarcinales* (other)	0.4-15.8	(Knittel et al. 2005)
					*Euryarchaeota* (other)	0.7	
					*Methanomicrobia* (other)	2.6	
ANME2c-622	CCC TTG GCA GTC TGA TTG	622-639	ANME-2c	76.2	*Methanosarcinales* (other)	3.7	(Knittel et al. 2005)
					*Methanomicrobia* (other)	2.2	
					ANME-2a/b	2.5	
ANME-2c-760	CGC CCC CAG CTT TCG TCC	760-777	ANME-2c	86.9	*Archaeoglobi*	2.7-2.9	(Knittel et al. 2005)
					*Methanosarcinales* (other)	0.6-5.3	
					ANME-2a/b	4.9	
					*Methanomicrobia* (other)	2.9	
					*Ca.* Methanoperedens	1.2	
					GOM-Arc I	0.9	
					ANME-3	1.5	
					ANME-1b	0.7	
					*Euryarchaeota* (other)	0.7	
EelMS240	CCC ACT ACA ACC TGA TAG	240-257	ANME-2a/b	79.5	*Methanosaetaceae*	4.5	(Boetius et al. 2000)
			ANME-2c	60.8	*Thermoplasmata*	0.1-50	
					*Methanosarcinaceae* (other)	6.9-73	
			ANME-3	27.3	*Methanomicrobiales* (other)	0.2-5.9	
					*Thermococci*	0.4	
			ANME-2b	83.3	GOM Arc I	14	
					*Ca.* Methanoperedens	12.2	
					*Methanosarcinales* (other)	1-56.7	
EelMS538	CGG CTA CCA CTC GGG CCG C	538-556	ANME-2c	89.2	GOM Arc I	54.8	(Boetius et al. 2000)
					*Ca.* Methanoperedens	67.5	
			ANME-2a/b	61.5	*Methanosaetaceae*	0.1	
					Candidate division MSBL1	9.1	
			ANME-2b	12.5	*Methanosarcinaceae* (other)	1-31.6	
					*Methanomicrobia* (other)	1-7.2	
EelMS932	AGC TCC ACC CGT TGT AGT	932-949	ANME-2b	12.5	*Methanosarcinales* (other)	0.7-9.7	(Boetius et al. 2000)
			ANME-2c	6	*Methanococcales*	1.1-10	
			ANME-1	4.8	*Archaeoglobi*	1.3-4.5	
			ANME-3	1.5	*Methanomicrobia* (other)	1-2.7	
			ANME-2a/b	0.8	GOM Arc I	0.9	
					*Ca.* Methanoperedens	1.2	
ANME-2b-729	CGTTCTCGTAGGGCGCCT		ANME-2b	75	*Methanosarcinales* (other)	0.4	(Hatzenpichler et al. 2016)
			ANME-2a/b	5.7	*Methanomicrobia* (other)	0.1	
					*Euryarchaeota* (other)	0.1	

All probes were tested using the online Testprobe database of SILVA, using 100% specificity (0 mismatches). Probes tested in this study are given in bold

### Environmental samples and pure cultures

Samples were taken from Eckernförde Bay (Baltic Sea) at station B (water depth 28 m; position 54° 31′ 15 N, 10° 01′ 28 E) during a cruise of the German research vessel *Littorina* in June 2005. This sampling site has been described by Treude et al. (2005b). Sediment samples were taken with a small multicore sampler as described previously (Barnett et al. 1984). The cores had a length of 50 cm and reached 30–40 cm into the sediment bed. Immediately after sampling, the content of the cores was mixed in multiple large bottles, which were made anoxic by replacing the headspace with anoxic artificial seawater. In the laboratory, the headspace was replaced by CH_4_ (0.15 MPa) and bottles were kept at 4 °C in the dark. *M. mazei* strain MC3 (DSM**-**2907) and *Desulfovibrio* G11 (DSM-7057) were obtained from the culture collection (DSMZ, Braunschweig, Germany).

### DNA isolation

Genomic DNA was extracted using the Fast DNA Kit for Soil (MP Biomedicals, Solon, OH) according to the manufacturer’s protocol with two 45-s beat beating steps using a Fastprep Instrument (MP Biomedicals, Solon, OH). Afterwards, DNA was purified and concentrated using the DNA Clean & Concentrator kit (Zymo Research Corporation, Irvine, CA). The DNA concentrations were either determined with the NanoDrop® ND-2000 (Thermo Fisher Scientific, Waltham, MA) or the Qubit 2.0 fluorometer (Thermo Fisher Scientific).

### Quantitative real-time PCR

PCR amplifications were done in triplicate in a BioRad CFX96 system (Bio-Rad Laboratories, Hercules, CA) in a final volume of 25 μl using iTaq Universal SYBR Green Supermix (Bio-Rad Laboratories), 5 μl of template DNA, and 1 μl of forward and reverse primers (concentration of 10 μM), all according to the manufacturer’s recommendations. Triplicate standard curves were obtained with tenfold serial dilutions ranging from 2 × 10^5^ (corresponding to 1 ng μl^−1^ DNA) to 2 × 10^−2^ copies per microliter of plasmids containing 16S rRNA archaeal inserts of ANME-1 (HP-Arch-D10, Genbank ID: HF922261.1), ANME-2a/b (HP-Arch-B12, Genbank ID: HF922244.1), and ANME-2c (HP-Arch-F07, Genbank ID: HF922279.1). All used primers were extensively tested for specificity with cloned archaeal inserts of ANME-1, ANME-2a/b, ANME-2c, *Methanococcoides* sp. (HP-Arch-F02, Genbank ID: HF922275.1), and genomic DNA of *M. mazei* strain MC3 (DSM-2907) and *Desulfovibrio* G11 (DSM-7057), as well as with a complex environmental sample from Eckernförde Bay (EB0). For most primer sets, the first strategy was to reproduce PCR conditions as described in the original literature. When not satisfactory, annealing temperatures were optimized by performing a gradient PCR using all of the above listed test samples. Primers specific for amplification of ANME-1, ANME-2a/b, and ANME-2c archaea were validated. After amplification, specificity was checked by performing a melting curve analysis. This consisted of a temperature gradient (72–95 °C) to obtain the specific melting temperature of the PCR products. PCR products with a different sequence and size will show a different melting temperature. Melting curve analysis of PCR products gives an accurate and sensitive measurement of the amount and the difference of the PCR products that were formed as compared to the positive control. Afterwards, PCR products were also checked for the correct size on a 1.5% agarose gel, using the 1-kb plus ladder as size reference (Thermo Scientific).

## Results

### In silico testing of probes and primers

In silico probe and primer matching was done with published probes and primers to obtain coverage and specificity of target groups (marine ANME subclades 1, 2a/b, and 2c) and non-target groups, allowing zero mismatches (100% specificity). In Table 1, results of the primer matching (i.e., in silico PCR) are shown for all primer pairs used in previous studies. Most primer pairs showed a good coverage of the target group with little coverage of non-target groups. Only primer pair ANME-2aF/ANME-2aR did not have a specific target and primer pair ANMEF/907R only targeted a small fraction of ANME-3. The results of probe matching, which does not match primer pairs, but matches single oligonucleotide sequences to the SILVA 16S rRNA gene database, are given in Table 2. These results show that a significant amount of probes show zero mismatches with non-target groups, sometimes with a high coverage. Primer pairs with highest target group coverage and least non-target group coverage were tested in vitro using quantitative PCR (qPCR) and are given in bold in Table 1.

### In vitro testing of primers

#### ANME-1

ANME-1-337F and ANME-1-724R (Girguis et al. 2005) showed highest coverage of the target group, with lowest coverage of non-target groups in the in silico analysis (Table 1). This primer pair was described to be specific for ANME-1, had strong 3′-mismatches to closely related outgroups, and was previously tested for amplification with *Desulfobulbus* spp., *Beggiatoa* spp., and 28 archaeal and bacterial phylotypes commonly found in seep sediments (Girguis et al. 2005). Here, specificity was tested using qPCR with genomic DNA of *M. mazei* strain MC3 and cloned full-length 16S rRNA gene sequences of ANME-1 and ANME-2c as DNA template. This revealed that the ANME-1 primer pairs were not specific under described reaction conditions. The ANME-1 primer pair gave a PCR product with genomic DNA of *M. mazei* strain MC3 as DNA template consisting of two bands, with one having the correct fragment size of 358 bp for this primer set. Melting curve analysis showed that (one of the) PCR products also had an identical melting temperature compared to the PCR product of the positive control. The primer pair also gave multiple PCR products with the cloned 16S rRNA gene sequence of ANME-2c as template DNA, with none of these products having the expected amplicon size of 358 bp (Fig. S1). This also counted for the cloned 16S rRNA gene sequence of ANME-2a/b.

**Fig. 3:**
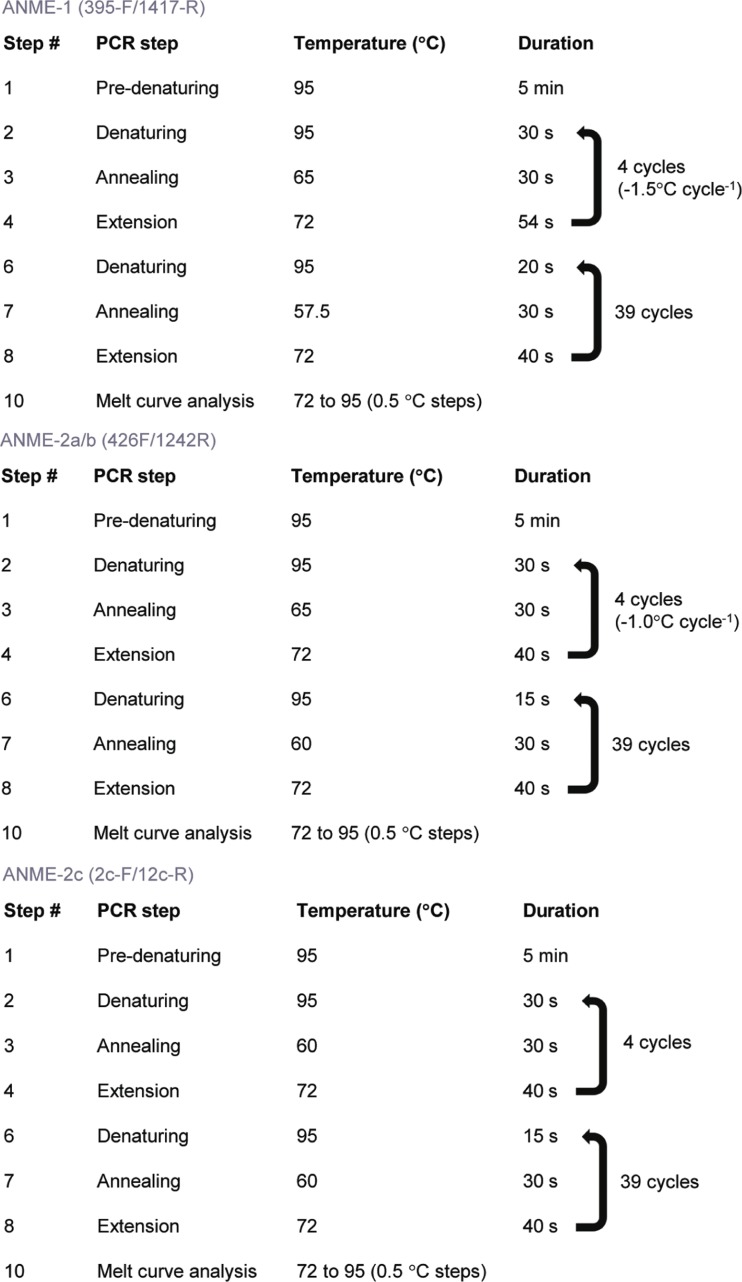
Optimized qPCR programs for all archaeal primer sets used in this study

Another primer pair was described to be specific for ANME-1: ANME1-395F and ANME1-1417R) (Miyashita et al. 2009). With these designed primers for ANME-1, Miyashita et al. (2009) tested the specificity using genomic DNA from *Methanogenium organophilum* and *Methanomicrobium mobile*. Detection of ANME in methanogenic environments such as methanogenic sludge, rice field soils, lotus field sediment, and natural gas fields was also performed (Miyashita et al. 2009). However, under the reported conditions that were applied to our Eckernförde Bay samples, the PCR efficiency with the ANME-1 primers was only 61.8% and the calibration curve showed an *R* ^2^ value of only 0.973. After optimization, mainly changing annealing temperatures, these values greatly improved (efficiency = 87%, *R* ^2^ = 0.998) and melting temperatures of PCR products from both the cloned 16S rRNA gene sequence of ANME-1 and from the Eckernförde Bay environmental sample EB0 were identical (Fig. S2). For the ANME-1 primer set, genomic DNA from *M. mazei* strain MC3 and *Desulfovibrio* G11 as template DNA did not give a PCR product after optimization. Only when using template concentrations of >2 × 10^2^ 16S rRNA gene copies μl^-1^ cloned 16S rRNA gene sequences of ANME-2a/b and ANME-2c as DNA template gave a PCR product (Figs. S3 and S4). Furthermore, when this cloned ANME-2c 16S rRNA gene sequence gave a PCR product, the melting temperature was not the same as for the cloned ANME-1 16S rRNA gene sequence and sample EB0 as template DNA and the PCR product(s) were not of the expected size of 1039 bp (Fig. S4). Although the efficiency of the primer set was not high, probably due to the length of the PCR product (efficiency should be between 90 and 100% and product length is optimal between 70 and 200 bp), these primers seem to be specific and appropriate for quantification using our protocol (Fig. 3), but the low efficiency may result in low sensitivity when target concentrations are low.

#### ANME-2a/b

For specific detection of the coherent clade ANME-2a/b, primer set ANME-2a-426-F and ANME-2a-1242-R (Miyashita et al. 2009) were tested in this work. Amplification of the cloned 16S rRNA gene sequence of ANME-1 as DNA template only occurred at concentrations of >2 × 10^1^ 16S rRNA gene copies μl^-1^, and the PCR product showed a different melting temperature at lower template DNA concentrations. Only at higher template concentrations of ANME-1 cloned sequences of >2 × 10^2^ 16S rRNA gene copies μl^-1^, the PCR products were visible (Fig. S5). Cloned ANME-2c 16S rRNA gene sequences as template DNA for this ANME-2a/b primer pair only showed a PCR product at concentrations of >2 × 10^2^ 16S rRNA gene copies μl^-1^ as seen from the melting curve analysis, but the product quantity was too low for a visible product on an agarose gel (Fig. S6). The same result was observed with cloned *Methanococcoides* sp. 16S rRNA gene sequences as template DNA. The Eckernförde Bay sample as template DNA resulted in a PCR product with a melting temperature that corresponded to the PCR product of the cloned ANME-2a/b 16S rRNA gene sequence as template DNA. Since the environmental sample EB0 used in this study has a low amount of the ANME-2c subtype (Timmers et al. 2015a, b), this protocol can be applied for this specific sample (Fig. 3). Although the coverage of this primer set is not optimal (±38%), other published ANME-2a/b primer sets were not sufficiently covering the target groups (Table 1).

#### ANME-2c

The primer pair AR468f and AR736r was described to be specific for ANME-2c and has been tested for specificity with *Methanosarcina acetivorans* and other representative archaeal groups commonly found in seep sediments (Girguis et al. 2003). The primers showed a high coverage of target groups with low coverage of non-target groups (Table 1). However, when we performed qPCR, the primer pair was not specific under described reaction conditions. It showed a PCR product with template DNA from *M. mazei* strain MC3, and the amplified product had the same expected product size of 268 bp and the same melting temperature as the positive control (Fig. S7). This was also the case for the cloned ANME-1, ANME-2a/b, and ANME-2c 16S rRNA gene sequence.

The forward primer AR468f was also used in a mixture of three separate forward primers to increase coverage, together with a new reverse primer ANME-2c-AR-1411R (Miyashita et al. 2009). This primer pair indeed showed higher coverage of the target group with low coverage of non-target groups (Table 1). This primer pair has been tested for specificity using genomic DNA from *Methanogenium organophilum* and *Methanomicrobium mobile* (Miyashita et al. 2009). Detection of ANME in methanogenic environments such as methanogenic sludge, rice field soils, lotus field sediment, and natural gas fields has also been performed, as was done for the ANME-1 primers (Miyashita et al. 2009). In our experiments, the primer set showed a PCR product with genomic DNA from *M. mazei* strain MC3 and *Desulfovibrio* G11 as well as with all cloned ANME-1 and ANME-2a/b 16S rRNA gene sequences as a DNA template. However, multiple PCR products were obtained, but none had the expected product size and melting temperatures. This was in contrast with PCR products of the cloned 16S rRNA gene sequence from ANME-2c as DNA template (Fig. S8). The authors claimed that it was indeed difficult to design primers perfectly specific for ANME-2c sequences (Miyashita et al. 2009).

Primers for ANME-2c were designed by others as well, such as ANME-2c-F and ANME-2c-R that showed highest coverage of the target group (Table 1) (Vigneron et al. 2013). Under described PCR conditions, ANME-1, ANME-2a/b, *Methanococcoides* sp., and all negative controls gave a PCR product of the expected size. However, after optimization, no PCR amplification of the cloned ANME-2a/b 16S rRNA gene sequence was observed, although ANME-2a/b was targeted with zero mismatches (Table 1). PCR amplification of the cloned 16S rRNA gene sequence of ANME-1 as DNA template only occurred with template concentrations of >2 × 10^2^ 16S rRNA gene copies μl^-1^ (Fig. S9). The PCR product of the Eckernförde Bay sample showed a melting temperature corresponding to the PCR products of the cloned ANME-2c 16S rRNA gene sequence. Eckernförde Bay samples have low copy numbers of the ANME-1 subtype, and therefore, this protocol can be used in these types of sediments. Although DNA of *M. mazei* strain MC3 and *Desulfovibrio* G11 did show a PCR product with these primers, the melting temperature did not correspond to the melting temperature of the PCR product of the cloned ANME-2c 16S rRNA gene sequence, which is reflected in the different PCR product size. Therefore, when using these primers for environmental samples, quantification of ANME-2c cannot be done when multiple PCR products are obtained and when different melting temperatures are obtained that are identical to those of *M. mazei* strain MC3. The optimized protocol for the ANME-2c specific primers is given in Fig. 3.

## Discussion

From all 16S rRNA gene-based published probes and primers that were so far designed to be specific for different ANME subtypes, many were not specifically targeting the clades that these probes and primers were designed for (Tables 1 and 2). The non-target 16S rRNA sequences that showed no mismatches, especially with the investigated probes, included some problematic non-targets which are shown in Table 2. These were other marine ANME clades and the GoM-Arc I clade, also known as the AAA archaea (Knittel and Boetius 2009). This clade contains the recently described “*Candidatus* Methanoperedens nitroreducens” that belongs to the ANME-2d subclade that coupled AOM to nitrate and iron or manganese reduction (Raghoebarsing et al. 2006; Haroon et al. 2013; Ettwig et al. 2016; Arshad et al. 2015). There is not much known on the occurrence and activity of the ANME-2d subclade and on the overarching GoM-Arc I/AAA clade. However, the GoM-Arc I/AAA clade has been found to co-occur with ANME types that are known to be involved in AOM coupled to sulfate reduction, such as ANME-1, ANME-2a/b, and ANME-2c (Timmers et al. 2016; Mills et al. 2003; Mills et al. 2005; Lloyd et al. 2006). Therefore, probes and primers specific for ANME involved in sulfate-dependent AOM should not match with 16S rRNA gene sequences of the GoM-Arc I/AAA clade, if this clade co-occurs with the ANME subtypes. With all ANME sequences in the SILVA 16S rRNA gene database version SSU r122 Ref NR (Quast et al. 2013), we calculated the similarity between and within ANME clades and the GoM-Arc I clade that contained ANME-2d, using the distance matrix method of the ARB software package with similarity correction (Ludwig et al. 2004). The lowest similarity was between ANME-1 and ANME-2c clades and was 74.2%, which is lower than previously reported by Knittel and Boetius (2009) (Fig. 1). The lowest similarity within ANME clades was within ANME-1 and was only 80.6% (Fig. 1). Since this inter- and intra-group diversity is high, designing primers that should specifically target ANME subclades without targeting outgroups is deemed difficult. New sequences added to the database can also drastically change coverage and specificity of previously designed probes and primers, and therefore, probe and primer validation needs to be reconsidered constantly. As an alternative to (or complement with) the 16S rRNA gene as biomarker, one can use functional marker genes such as the gene for the alpha subunit of the methyl coenzyme m reductase (*mcrA*) that is present in all methanogens and ANME subtypes. This *mcrA* gene is highly conserved, and comparative phylogenetic studies have clearly shown that *mcrA* and 16S rRNA gene-based phylogeny is consistent (reviewed in Friedrich (2005)). However, it has been recently discovered that not only methanogenic archaea possess the *mcrA* gene (Laso-Pérez et al. 2016).

After in silico selection of only 16S rRNA gene targeting primers and probes with highest coverage of target group and lowest coverage of non-target groups, we found that some of the selected primers and probes that should specifically target different ANME clades were not specific in our in vitro qPCR analysis. Most of these primers and probes can therefore not be applied to complex microbial communities where different ANME clades and methanogens co-occur, which is the case in most marine sediments. Validation with sequences from the in situ archaeal community of the new environment is therefore mandatory, or when no data on the archaeal community is available, one needs to be sure that the primers and probes used do not target close relatives and are specific.

After validation and optimization of published 16S rRNA gene targeting primer sets, we found three sets suitable for specific and quantitative detection of ANME-1, ANME-2a/b, and ANME-2c subclades in a complex marine environment, Eckernförde Bay, where different ANME subtypes and methanogens co-occur. The primer pairs ANME1-395F/ANME1-1417R, 426F/1242R, and 2c-F/12c-R were specific for detection of ANME-1, ANME-2a/b and ANME-2c, respectively and could be applied to other complex methanotrophic, and possibly methanogenic communities, with certain limitations. Obviously, described PCR conditions cannot be applied to other complex samples and plasmid 16S rRNA gene inserts and need to be validated every time. For the evaluation and optimization of the qPCR primers that was performed in this work, we developed and applied an operating procedure of which we believe should be followed when new samples with complex archaeal communities are obtained (Fig. 2):

1. Consult the literature for developed primers or design new primers. Perform in silico PCR to check the coverage of target and non-target groups or check the binding specificity of both forward and reverse primers.
2. Perform a gradient qPCR with a range around the obtained/described melting temperature (± −5/+5 °C), using suitable positive and negative controls to obtain the optimal annealing temperature.
3. Analyze the melting curves and use the annealing temperature that shows as little amplification with negative controls as possible, especially with close relatives and sequences know to be abundant in the samples.
4. When the melting curve is the same between target and non-target, perform qPCR with a DNA concentration gradient (tenfold dilutions) of positive (target) control and negative (non-target) control samples to determine at which concentrations amplification of the negative controls starts. When the quantity of the non-target in the samples is below the threshold concentration where amplification of the non-target starts, one can apply the primer set for the target. The positive (target) control DNA concentration gradient PCR results are used as a calibration curve to obtain the slope (*R* ^2^ > 0.99%) and primer efficiency (optimally between 90 and 100%).
5. When the melting curve of the target is different than from the non-target, the primers can still be used (obviously only with good efficiency and *R* ^2^), but only when the melting curve of the sample is the same as for the target. Also, no multiple melting curves and thus multiple PCR products should be observed. If so, the non-target may have been amplified. Moreover, melting curves of non-target DNA could change with changing concentration of template and therefore a concentration gradient of DNA is also advisable (Fig. 2, gray line).

## Electronic supplementary material

ESM 1 (PDF 721 kb)
