# Supplementary material for: Evaluation and optimization of PCR primers for selective and quantitative detection of marine ANME subclusters involved in sulfate-dependent anaerobic methane oxidation
Source: Appl Microbiol Biotechnol. 2017 Jun 15;101(14):5847–59. doi: 10.1007/s00253-017-8338-x (PMC5501906; doi:10.1007/s00253-017-8338-x)
Supplement: Supplementary file 1 — (PDF 721 kb) [file 253_2017_8338_MOESM1_ESM.pdf]

Evaluation and optimization of PCR primers for selective and quantitative detection of marine ANME subclusters involved in sulfate-dependent anaerobic methane oxidation

Applied Microbiology and Biotechnology, Supplementary material

Peer H.A. Timmers[a,b]#, H. C. Aura Widjaja-Greefkes[a], Caroline M. Plugge[a,b] and Alfons J. M. Stams[a,c]

[a]Wageningen University, Laboratory of Microbiology, Stippeneng 4, 6708 WE Wageningen, the Netherlands

[b]Wetsus, centre of excellence for sustainable water technology, Oostergoweg 9, 8911 MA Leeuwarden, the Netherlands

[c]Centre of Biological Engineering, University of Minho, Campus de Gualtar, 4710-057 Braga, Portugal.

#Corresponding author. Mailing address: Wageningen University, Laboratory of Microbiology, Stippeneng 4, 6708 WE Wageningen, Phone: +31 317483739. Fax: +31 317 483829. Email: peer.timmers@wur.nl

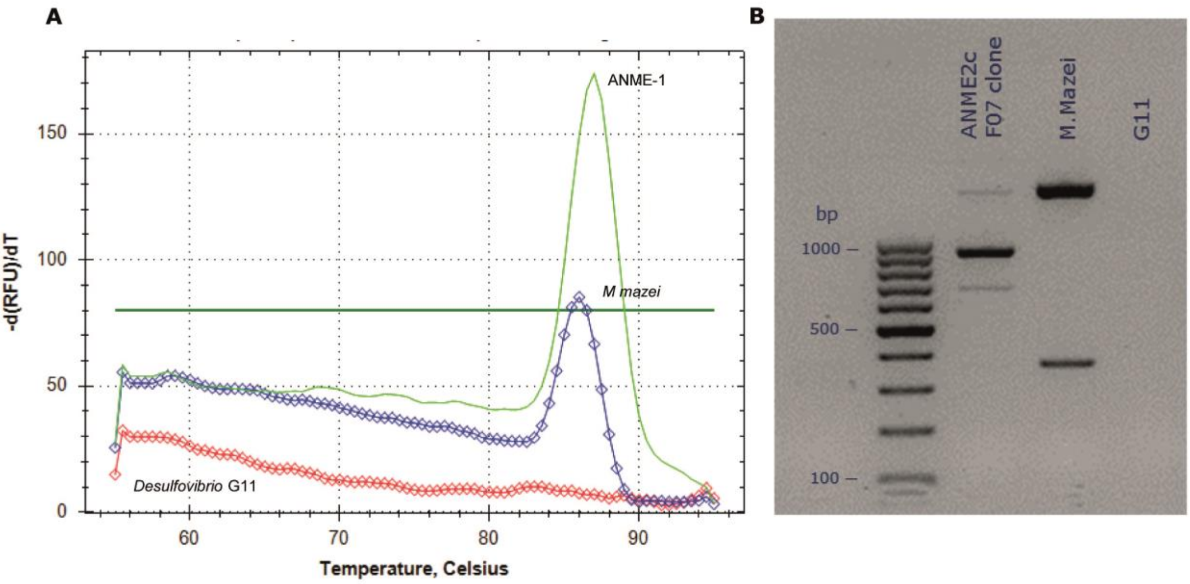

Fig. S1 A) Melting curve of the amplicon of ANME-1 primer pair ANME-1 337F and ANME-1 724R with *M. mazei* strain MC3 (purple diamonds, 1 ng µl^{-1} DNA), *Desulfovibrio* G11 (red diamonds, 1 ng µl^{-1} DNA) and cloned ANME-1 insert (yellow line, 2x10^{3} copies ul^{-1}). **B)** Agarose gel showing PCR products (expected product size of 358 bp) of ANME-2c cloned insert (2x10^{3} copies ul^{-1} in PCR) and products of *M. mazei* strain MC3 and *Desulfovibrio* G11(1 ng ul^{-1} DNA in PCR). The green line indicates the threshold line for quantification.

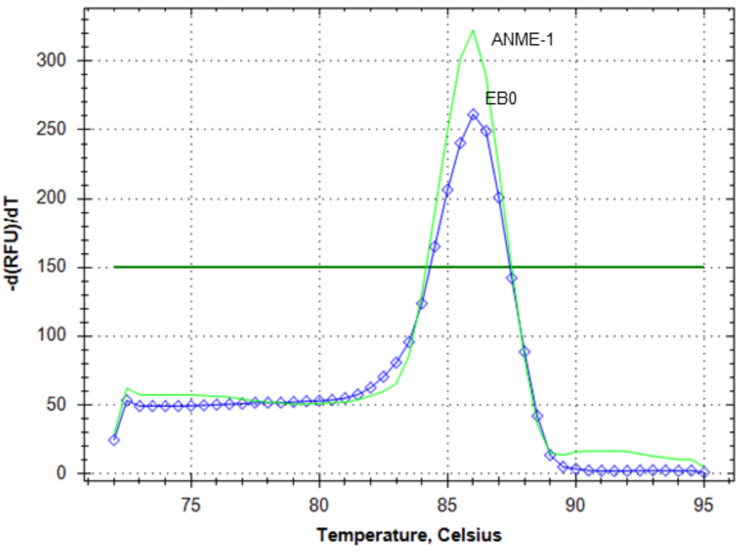

Fig. S2 Melting curve of the amplicon of ANME-1 primer pair ANME-1 395f and ANME-1 1417r with the optimized protocol for the Eckernförde bay sample (blue diamonds, 1 ng ul^{-1} DNA) and cloned ANME-1 insert (yellow line, $2x10^3$ copies ul^{-1}). The green line indicates the threshold line for quantification.

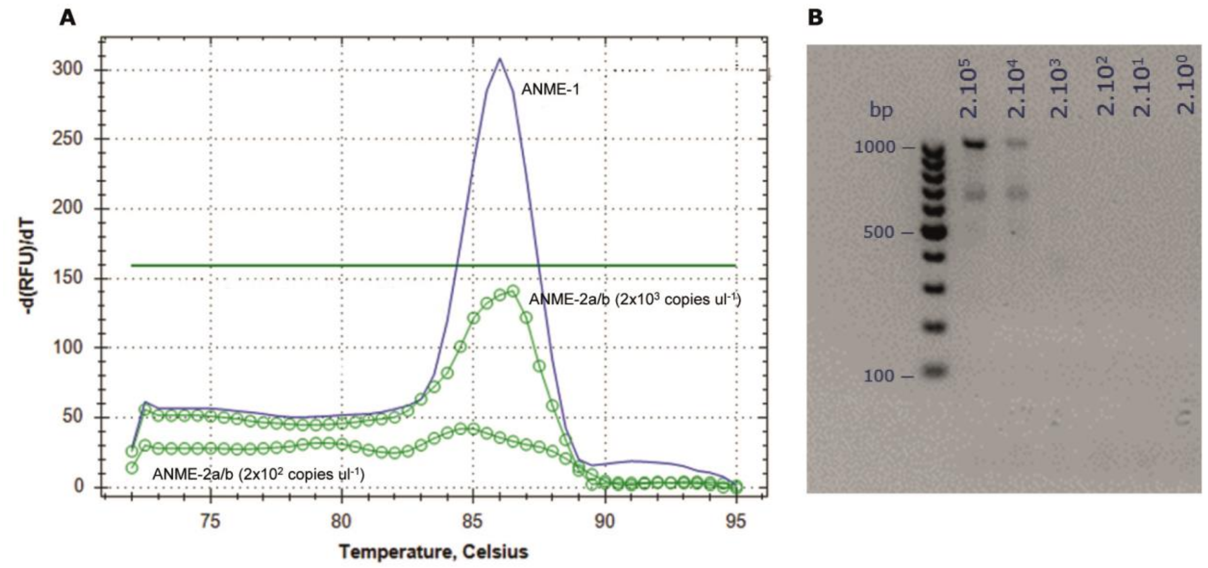

Fig. S3 A) Melting curve of the amplicon of ANME-1 primer pair ANME-1 395f and ANME-1 1417r with cloned ANME-1 insert (purple line, 2x10^{3} copies ul^{-1}), cloned ANME-2a/b insert (green circles: 2x10^{3} copies μl^{-1} and red circles: 2x10^{2} copies μl^{-1}). **B)** Agarose gel showing products (expected product size of 1039 bp) of ANME-2a/b cloned inserts with different concentrations (copies μl^{-1}) in the reaction mix. The green line indicates the threshold line for quantification.

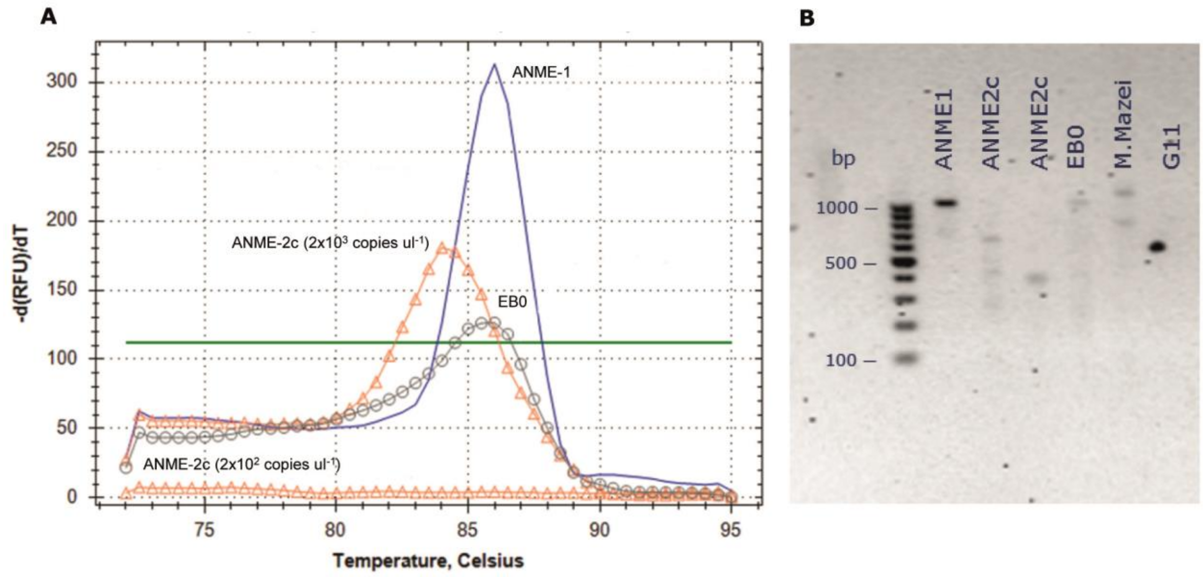

Fig. S4 A) Melting curve of the amplicon of ANME-1 primer pair ANME-1 395f and ANME-1 1417r with cloned ANME-1 insert (purple line, 2x10^3 copies µl^{-1}), cloned ANME-2c insert (triangles, 2x10^2 copies µl^{-1} and 2x10^3 copies µl^{-1}) and the Eckernförde bay sample (EB0, circles, 1 ng ul^{-1} DNA). **B)** Agarose gel showing products (expected product size of 1039 bp) of ANME-1 cloned insert, both ANME-2c cloned inserts, the Eckernförde bay sample (EB0), *M. mazei* strain MC3 and *Desulfovibrio* G11 (1 ng ul^{-1} DNA in PCR). The green line indicates the threshold line for quantification.

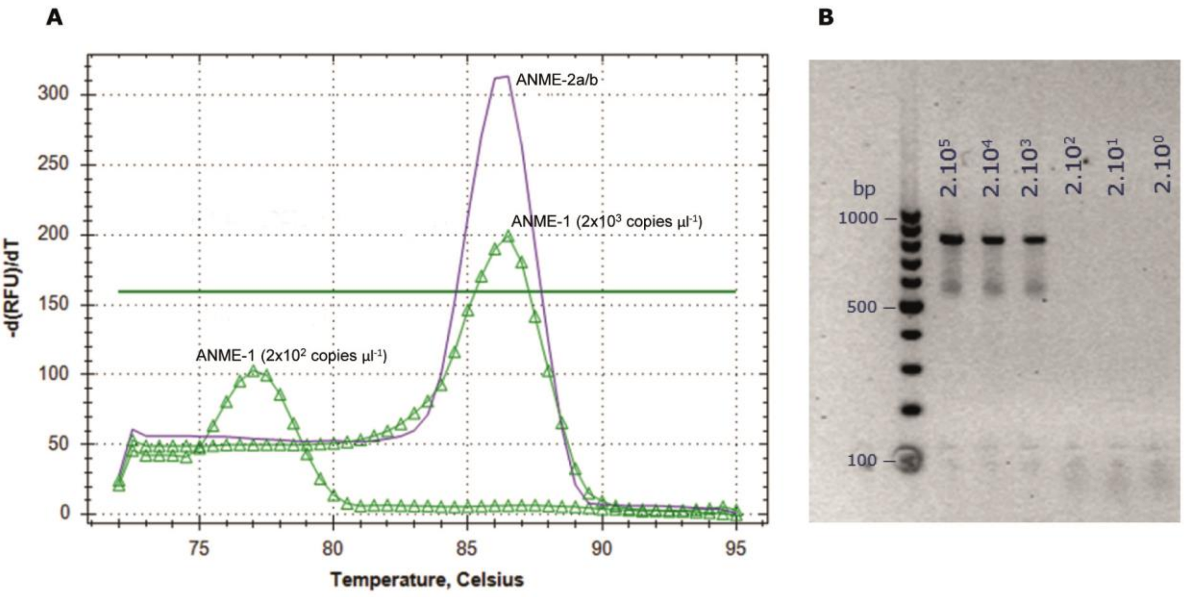

Fig. S5 A) Melting curve of the amplicon of ANME-2a/b primer pair ANME-2a-426-F and ANME-2a-1242-R with cloned ANME-2a/b insert (purple line, 2x10^3 copies μl^{-1}), cloned ANME-1 insert (triangles, 2x10^2 copies μl^{-1} and 2x10^3 copies μl^{-1}). **B)** Agarose gel showing products (expected product size of 833 bp) of ANME-1 cloned inserts with different concentrations (copies μl^{-1}) in the reaction mix. The green line indicates the threshold line for quantification.

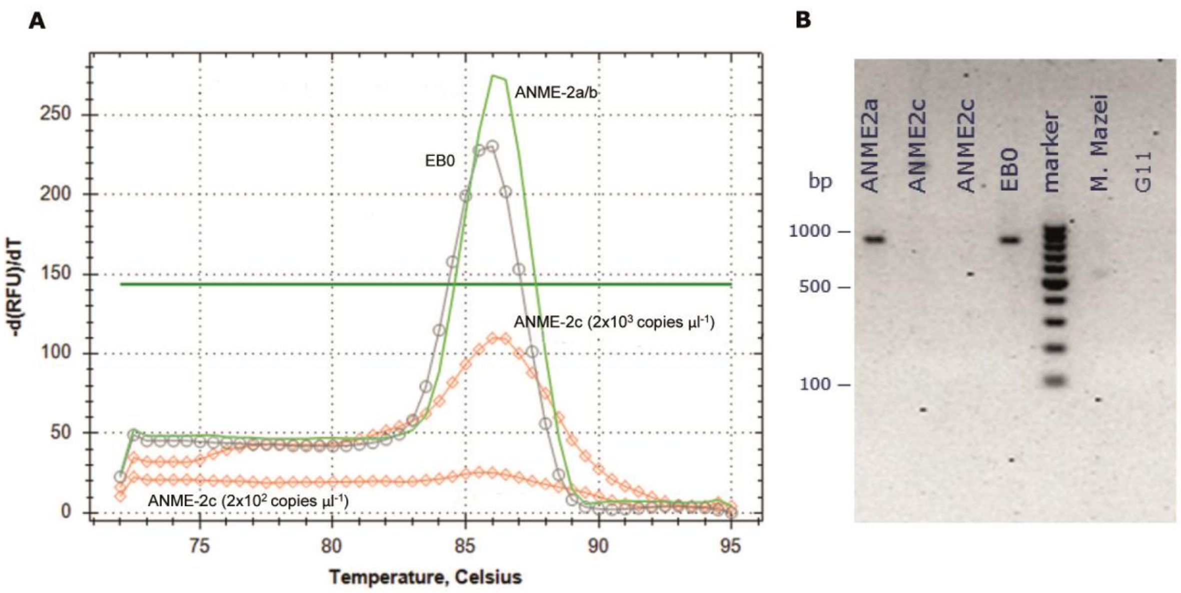

Fig. S6 A) Melting curve of the amplicon of ANME-2a/b primer pair ANME-2a-426-F and ANME-2a-1242R with cloned ANME-2a/b insert (orange line, $2x10^3$ copies μl^{-1}), cloned ANME-2c insert (diamonds, $2x10^2$ copies μl^{-1} and $2x10^3$ copies μl^{-1}) and the Eckernförde bay sample (EB0, circles, 1 ng ul^{-1} DNA). **B)** Agarose gel showing products with expected product size of 833 bp of ANME-2a/b cloned inserts, both ANME-2c cloned inserts, the Eckernförde bay sample (EB0), *M. mazei* strain MC3 and *Desulfovibrio* G11 (1 ng ul^{-1} DNA in PCR). The green line indicates the threshold line for quantification.

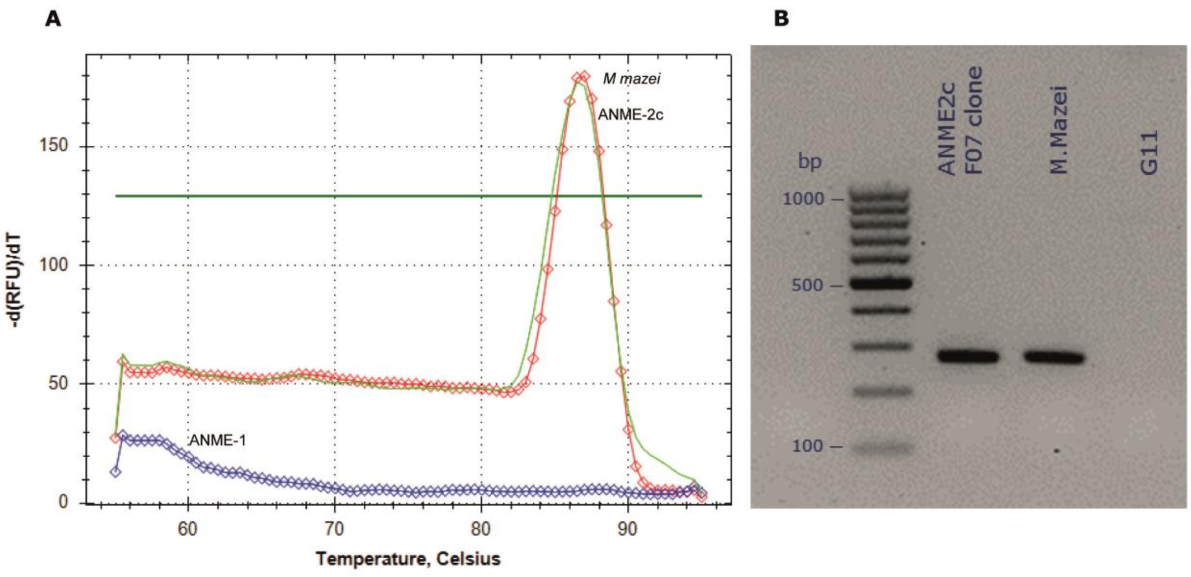

Fig. S7 A) Melting curve of the amplicon of ANME-2c primer pair AR-468f/AR-736r with *M. mazei* strain MC3 (red diamonds, 1 ng μl^{-1} DNA) and cloned ANME-1b insert (purple diamonds, ($2x10^3$ copies ul^{-1}) and cloned ANME-2c insert (yellow line, $2x10^3$ copies ul^{-1}). **B)** Agarose gel showing products (expected product size of 268 bp) of ANME-2c cloned insert and products of *M. mazei* strain MC3 and *Desulfovibrio* G11 (1 ng ul^{-1} DNA in PCR). The green line indicates the threshold line for quantification.

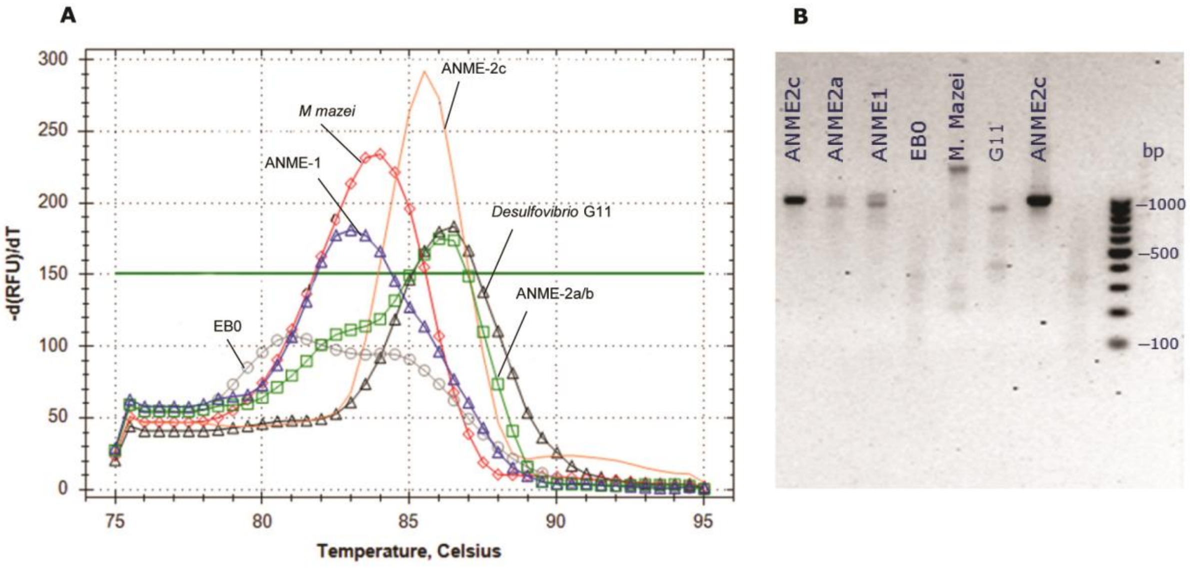

Fig. S8 A) Melting curve of the amplicon of ANME-2c primer pair AR468f and ANME-2c-1411R with cloned ANME-2c insert (yellow line, $2x10^3$ copies μl^{-1}), cloned ANME-1 insert (purple triangles, $2x10^3$ copies μl^{-1}), cloned ANME-2a/b insert (orange squares, $2x10^2$ copies μl^{-1}), the Eckernförde bay sample (grey circles, EB0, 1 ng ul^{-1} DNA), *Desulfovibrio* G11 (brown triangles, 1 ng ul^{-1} DNA) and *M. mazei* strain MC3 (orange diamonds, 1 ng ul^{-1} DNA). **B)** Agarose gel showing no products (expected product size of 960 bp) of ANME-2c, ANME-2a/b and ANME-1 cloned insert, the Eckernförde bay sample (EB0), *M. mazei* strain MC3 and *Desulfovibrio* G11. The green line indicates the threshold line for quantification.

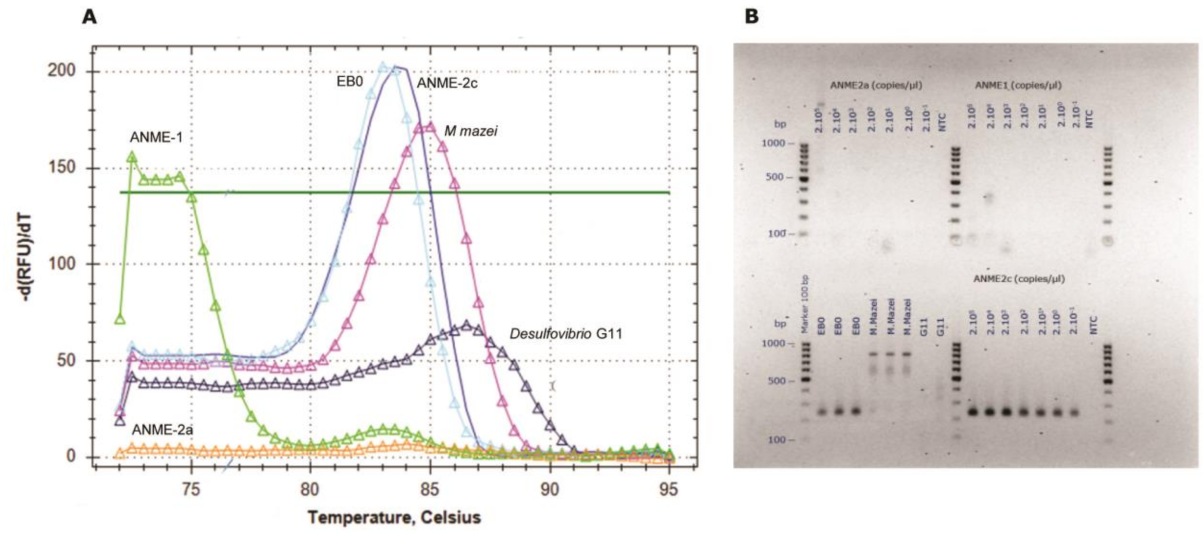

Fig. S9 A) Melting curve of the amplicon of ANME-2c primer pair 2c-F and 2c-R with cloned ANME-2c (purple line) and ANME-1 insert (orange triangles, $2x10^3$ copies μl^{-1}), the Eckernförde bay sample (EB0, light-blue triangles), *Desulfovibrio* G11(purple triangles) and *M. mazei* strain MC3(pink triangles) (1 ng ul^{-1} DNA). **B)** Agarose gel showing products (expected product size of 221 bp) of ANME-2a/b, ANME-1 and ANME-2c cloned insert with different concentrations (copies ul^{-1}), the Eckernförde bay sample (EB0), *M. mazei* strain MC3 and *Desulfovibrio* G11. The green line indicates the threshold line for quantification.
